# Biopsy of Human Morula-Stage Embryos: Outcome of 215 IVF/ICSI Cycles with PGS

**DOI:** 10.1371/journal.pone.0106433

**Published:** 2014-09-05

**Authors:** Elena E. Zakharova, Victoria V. Zaletova, Alexander S. Krivokharchenko

**Affiliations:** Center for Reproductive Medicine MAMA, Moscow, Russian Federation; The Chinese University of Hong Kong, Hong Kong

## Abstract

Preimplantation genetic diagnosis (PGD) is commonly performed on biopsies from 6–8-cell-stage embryos or blastocyst trophectoderm obtained on day 3 or 5, respectively. Day 4 human embryos at the morula stage were successfully biopsied. Biopsy was performed on 709 morulae from 215 ICSI cycles with preimplantation genetic screening (PGS), and 3–7 cells were obtained from each embryo. The most common vital aneuploidies (chromosomes X/Y, 21) were screened by fluorescence *in situ* hybridization (FISH). No aneuploidy was observed in 72.7% of embryos, 91% of those developed to blastocysts. Embryos were transferred on days 5–6. Clinical pregnancy was obtained in 32.8% of cases, and 60 babies were born. Patients who underwent ICSI/PGS treatment were compared with those who underwent standard ICSI treatment by examining the percentage of blastocysts, pregnancy rate, gestational length, birth height and weight. No significant differences in these parameters were observed between the groups. Day 4 biopsy procedure does not adversely affect embryo development *in vitro* or *in vivo*. The increased number of cells obtained by biopsy of morulae might facilitate diagnostic screening. There is enough time after biopsy to obtain PGD results for embryo transfer on day 5–6 in the current IVF cycle.

## Introduction

Preimplantation genetic diagnosis (PGD) and preimplantation genetic screening (PGS) are routine procedures performed in many in vitro fertilization (IVF) clinics. In patients with genetic or chromosomal abnormalities, PGD is an integral part of the IVF program. The first report describing successful biopsy of the human embryo for PGD was performed in 3-day-old embryos, which consisted of 6–8 cleavage-stage cells [1], [2]. Presently, biopsies of 8-cell blastomeres are performed in IVF laboratories worldwide [3], [4], [5], [6]. However, it is more advantageous to biopsy the blastocyst trophectoderm on day 5 or 6 than cleavage-stage blastomeres on day 3 [7], [8], [9], [10]. Firstly, a greater amount of genetic material can be retrieved from biopsies of blastocysts than from cleavage-stage embryos [3], [11]. A greater number of cells facilitates genetic analysis, provides more accurate results, and helps to detect genetic and chromosomal abnormalities by the fluorescence *in situ* hybridization (FISH), polymerase chain reaction (PCR), and comparative genomic hybridization (CGH) methods [12]. Secondly, biopsy of the trophectoderm on day 5 post-fertilization involve embryos that have successfully passed the initial steps of cell differentiation (i.e., compaction and cavitation) during mammalian preimplantation development. Therefore, these embryos have the highest implantation potential [13], [14]. Thirdly, several recent studies also showed that the rate of aneuploidy is significantly lower in blastocysts than in cleavage-stage embryos [15], [16], [7], [12]. Lastly, a biopsy performed on cleavage-stage embryos is more damaging compared to one performed on blastocysts [17].

Despite these advantages, genetic screening of blastocysts is limited to several hours to a day before embryo transfer, which can result in the cancellation of embryo transfer during the current IVF cycle, blastocyst cryopreservation, and embryo transfer in the next cycle [18], [7], [19]. In addition, cells obtained from the trophectoderm by mechanical or laser resection are not always suitable for FISH because isolation and fixation of their nuclei might be complicated. While biopsies of cleavage-stage embryos or blastocyst trophectoderm are routinely performed for PGD, there are no published data on human morula-stage embryo biopsy on day 4. Here, we argue that compact morula-stage biopsy on day-4 has the same benefits as biopsy on day 5, and can be more clinically useful.

In this study, we present results from 215 IVF/ICSI cycles with PGS and morula-stage embryos biopsy. We also analyze data on percentage of blastocysts, pregnancy rates, birth delivery, and the child's health status after PGS.

## Materials and Methods

### Ethics statement

This study was approved by the institutional review board of the Center for Reproductive Medicine MAMA.

### Patients

A prospective cohort study was undertaken between September 2011 and February 2013 using the same approach as for the follow-up of IVF and ICSI children conceived in the same center. Each patient was randomly assigned into a treatment group (ICSI or ICSI/PGS). ICSI and ICSI/PGS groups did not differ significantly in age, and the patients were aged 33.8±3.9 and 34.4±4.2, respectively. PGS was performed on couples with poor embryo implantation after conventional ICSI, on infertile couples due to a male factor, and on couples with a history of recurrent miscarriages. All patients signed an informed consent form for ICSI or ICSI/PGS that included counseling on the IVF program, the risk of ovarian hyperstimulation syndrome, pregnancy probability, the risk of pregnancy complications, the necessity of a prenatal diagnosis, and the possible cryopreservation of supernumerary embryos obtained during the program. When requested by the patient, PGS was performed to detect the most common vital aneuploidies (chromosomes X/Y, and 21) [20].

### IVF procedure

Patients underwent ovarian stimulation using the short antagonist protocol with urinary hormones and recombinant follicle-stimulating hormone (FSH). The dose of FSH was adjusted individually according to the patient's ovarian response. Human chorionic gonadotropin (hCG; 10,000 IU) was administered when at least three follicles were ≥17 mm in diameter. Oocyte retrieval was performed 36 h after the administration of hCG by ultrasound-guided puncture of ovarian follicles. Oocytes at MII were microinjected with ejaculated spermatozoa. The embryos were cultured before and after biopsy using standard embryo culture conditions in our laboratory.

### Biopsy and diagnosis of morula-stage embryos

Embryos that reached the morula stage by day 4 after fertilization, without signs of vacuolization or fragmentation, were incubated in Ca2+-free biopsy medium for 15 min. After mechanical drilling of the zona pellucida, morula cells were retrieved using a technique similar to blastomere biopsy at the cleavage stage.

For genetic diagnosis, we used FISH, which was the most popular method of preimplantation diagnosis in Europe at the beginning of this study: 73,7% of PGD/PGS (4500/6102) were performed using FISH according to the ESHRE PGD Consortium data collection XII [5]. FISH was performed by first incubating in a 1% tri-sodium citrate hypotonic buffer before fixation in Carnoy's solution (3∶1 ratio of methanol to glacial acetic acid) and then mounting on poly-L-lysine-treated slides. Hybridization to chromosome 21 (21q22.13-22.2/LSI 21/Spectrum Orange), the Y-chromosome (SEP Y/DYZ1/Spectrum Aqua), and the X-chromosome (SEP X/DXZ1/Spectrum Green) was performed using fluorescently labeled DNA probes for 16–24 hours in a 37°C humidified chamber and positive signals were visualized with DAPI staining by fluorescent microscopy.

### Embryo transfer and follow-up

One or two embryos were transferred on day 5 or 6. Supernumerary unaffected embryos were cryopreserved. Pregnant patients were followed up until week 12, after which they were forwarded to other clinics specializing in pregnancy care. If agreed upon by the patient, a control prenatal diagnosis was performed. Nurses provided additional pregnancy care until childbirth. After delivery, patients were asked to provide information on the delivery date, and on the child's height, weight, and overall health status.

### Statistics

The mean, standard deviation, median, and quartiles of distribution were determined for each continuous variable. Shapiro-Wilk and Pearson's chi-squared tests were used to determine the distribution of different parameters. The Mann-Whitney U test was used to compare groups according to the key indicators. The Fisher's exact test was used to analyze the 2×2 contingency tables. The significance level was set at 0.05 for all statistical tests.

## Results

### Biopsy of morula-stage embryos: clinical and embryological data, the pregnancy rate, and delivery data

**Figure 1:**
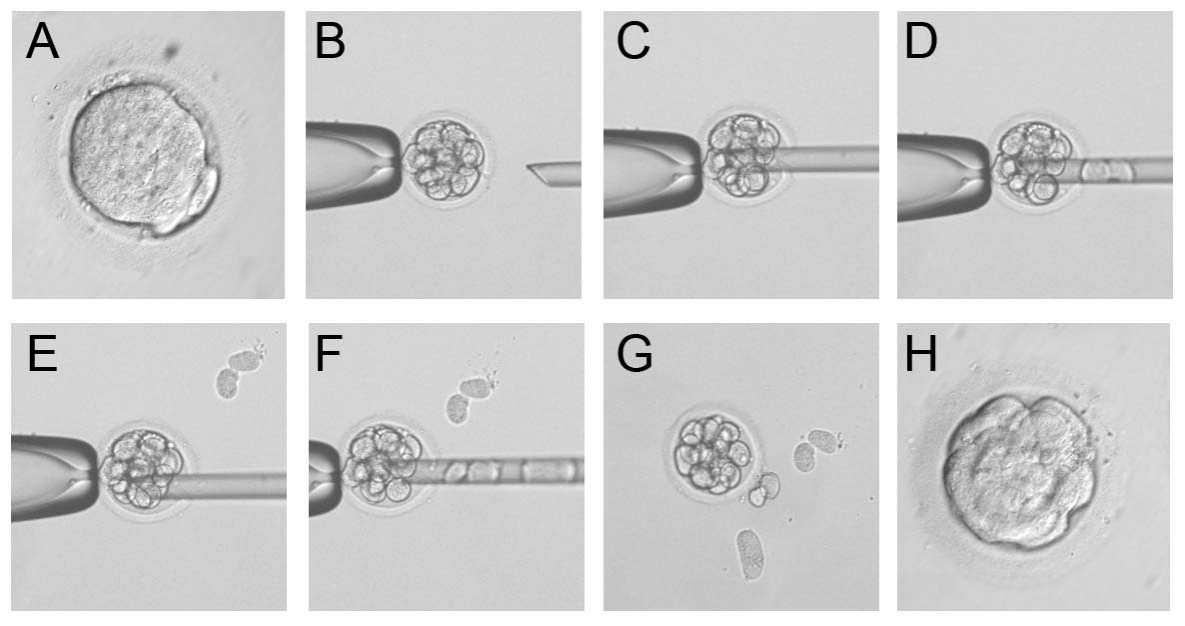
Biopsy of compact morula-stage embryos. (**A**) A compact morula-stage embryo before biopsy. (B–G) Steps of the biopsy. (H) An embryo at 2 h after biopsy.

A total of 709 morulae were biopsied from 215 ICSI/PGS cycles, and 3–7 cells were obtained from each embryo. Post-biopsy embryos were placed in culture medium supplemented with Ca2+, and cells restored intercellular contacts after 2–3 hours (Figure 1). A total of 645 post-biopsy embryos reached the blastocyst stage by day 5. In total, 396 blastocysts were transferred into the uterus in 208 ICSI cycles on day 5 (176/208) or day 6 (32/208) after fertilization, and 355 blastocysts were transferred into the uterus in 195 ICSI/PGS cycles on day 5 (168/195) or day 6 (27/195) after fertilization. Transfers were performed randomly on day 5 or 6, and the ratio was comparable between groups. There was no difference in the pregnancy rate after transfers on day 5 or 6 in both groups, or between groups. Clinical pregnancy was reached in 71 cases in the ICSI group and in 64 cases in the ICSI/PGS group. Table 1 presents embryological and clinical data from the ICSI and ICSI/PGS groups. Of 64 cases in the ICSI/PGS group, 52 pregnancies resulted in delivery, and 7 pregnancies are on-going at the time of writing of this study. Table 2 presents data on birth outcomes and the number of babies born in each group.

**Table 1:** Embryological and clinical data.

DATA	ICSI/PGS (n = 215)	ICSI (n = 224)	*P*-value
Female age (years)	34.4±4.2	33.8±3.9	0.14
COCs per OR	7.9±2.5	8.2±3.2	0.63
**Embryology**			
Fertilization rate (%)	79.5	82.3	0.06
Blastocysts formation rate (blastocysts/zygotes,%)	58.9	57.74	1.26
**Clinical outcome**			
Clinical pregnancy	64	71	
Clinical pregnancy rate (% per OR)	29.7	31.6	0.37
Clinical pregnancy rate (% per ET)	32.8	34.1	0.38
Implantation rate % (FHB/embryos transferred)	21.1	20.7	0.39
Miscarriage	7	9	
Miscarriage (%)	10.9	12.7	0.39

**Table 2:** ICSI/PGS and ICSI children born.

	Deliveries			Born			Stillborn			Alive		
	ICSI/PGS	ICSI	Total	ICSI/PGS	ICSI	Total	ICSI/PGS	ICSI	Total	ICSI/PGS	ICSI	Total
Singleton	43	44	**87**	43	44	**87**	1	1	**2**	42	43	**85**
Twins	9	8	**17**	18	16	**34**	0	0	**0**	18	16	**34**
Total	52	52	**104**	61	60	**121**	1	1	**2**	60	59	**119**

We compared several birth parameters, namely, gestational length and birth height and weight, between the ICSI and ICSI/PGS groups (Table 3). There were no significant differences in these parameters between groups.

**Table 3:** Mean gestational length, and birth weight and height in ICSI/PGS children compared with ICSI children.

	ICSI/PGS	ICSI	*P*-value
**Gestational length (weeks)**			
Singleton	38.5±2.3	38.6±2.2	0.79
Twins	35.1±3.9	35.6±2.5	0.90
**Birth weight (gram)**			
Singleton	3222±497	3303±539	0.44
Twins	2334±784	2262±528	0.48
**Birth height (cm)**			
Singleton	51.1±2.3	51.3±2.9	0.56
Twins	44.6±5.8	45.1±4.1	0.94

### FISH

**Figure 2:**
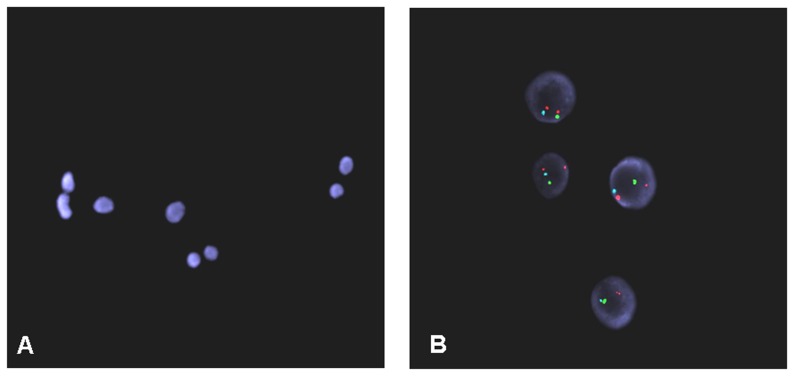
Cells obtained by biopsy for PGD. (A) Fixed nuclei stained with DAPI. (B) FISH signals for chromosome 21 (orange), the Y-chromosome (aqua), and the X-chromosome (green).

Aneuploidy was detected by FISH (chromosomes X/Y, 21) in 20.8% of embryos, with 79.2% of embryos showing no sign of aneuploidy (Figure 2). Results were inconclusive in 8.2% of embryos. Table 4 presents PGS cycle data.

**Table 4:** PGS cycle data.

FISH results	n	%
Biopsied (day 4, compact morula stage)	709	100
**Diagnosed**	**651**	**91,8**
No signal/inconclusive	58	8,2
**Normal**	**516**	**79,2**
**Abnormal**	**135**	**20,8**
Extra chromosome 21	26	19,2
Missing chromosome 21	9	6,6
Missing sex chromosome	17	12,6
Extra sex chromosome/chromosomes	39	28,9
Haploidy	10	7,4
Triploidy	16	11,8
Tetraploidy	9	6,6
Mosaicism	9	6,6

## Discussion

Genetic screening of embryonic cell DNA obtained from preimplantation embryos is routinely performed during IVF treatment. While biopsies of 6–8-cell embryos (blastomere biopsy) or blastocysts (trophectoderm biopsy) are generally successful [21], [22], compact morula stage biopsy is considered to be impossible because cells are closely compacted and adhesive to other cells making viable biopsy extremely difficult [23]. In mammals such as humans, cell-cell contacts during morula compaction are mediated by uvomorulin (E-cadherin), a transmembrane calcium-dependent cell adhesion glycoprotein anchored to the cytoskeleton through catenins [24], [25].

Artificial decompaction of murine morula was for the first time demonstrated by R.Pey et al. [26]. While artificial decompaction in Ca2+-free culture medium is possible, it might be the result of conformational changes only within the extracellular domain of E-cadherin. It does not disrupt the adhesive properties of E-cadherin, and new contacts form upon the addition of Ca2+ [26]. We showed that Ca2+-free culture medium induced decompaction of human morula-stage embryos, which facilitated successful biopsy for genetic screening. The loss of intercellular contacts in decompacted morulae allows clinicians to obtain several cells for genetic diagnosis. Recompaction occurred in all embryos by 2–3 h after the addition of Ca2+ into the culture medium.

Our results showed that 3–7 cells can be obtained from morula-stage embryos. The number of cells obtained was comparable to that obtained from biopsies of the trophectoderm, which allowed the collection of 2–6 cells [27], [9], [28], [29] and was far better than that obtained from biopsies of cleavage-stage embryos, which permitted the collection of only 1–2 cells [3], [11]. According to the current ESHRE PGD Consortium data collection XII, biopsies on cleavage-stage embryos have been performed in 83.3% (5085/6102) of PGD/PGS cycles in Europe, whereas only 0.1% of biopsies have been performed on blastocysts (6/6102) [5]. In light of these findings, the method of morula-stage biopsy described here is technically equivalent to cleavage-stage biopsy and it provides almost as many cells as the blastocyst biopsy.

While cells obtained from the trophectoderm are often damaged due to microsurgery or laser dissection, those obtained from morula-stage embryos are viable. Cell viability is crucial for FISH analysis because hypotonically-treated nuclei can be more easily isolated from cells with an intact cell membrane. According to the current ESHRE PGD Consortium data collection XII, 73.7% (5085/6102) of PGD/PGS have been performed by FISH [5], indicating that our method would be widely applicable. Additionally trophectoderm biopsies cannot always be used for FISH; instead, PCR-based PGD and CGH is used instead [30], [27], [9], [31], [32], [29]. Cells from morula-stage embryos can also be examined using PCR and CGH, which is an important consideration because these methods are growing in popularity.

The percentage of embryos that reach the blastocyst stage and the pregnancy rate are key evaluation criteria for human embryo development *in vitro* and *in vivo*. While several publications report the blastocyst yield to be 47–60% after biopsy of 3-day-old human embryos [4], [11], [9], we demonstrated that 91% of embryos reached the blastocyst stage to day 5 after biopsy of morula-stage embryos. This percentage is not significantly different from the blastocyst yield obtained during non-biopsied morula development in the ICSI group, which was 91.9% by day 5.

The pregnancy rate after biopsy of morula-stage embryos was 32.8%, which is not significantly different from that in the ICSI without PGS (34.1%). The absence of significant differences in blastocyst development or pregnancy rate indicates that the procedure had no negative impact on embryo development *in vitro* or *in vivo*. Sufficiently high rates of pregnancy and blastocyst formation, as well as reversible decompaction, were observed in 4-day-old biopsies; thus, this method is efficient and safe for use on morula-stage embryos. Although studies of human morula-stage embryo biopsies have not been reported, Krzyminska and colleagues showed that compact morula stage biopsy significantly reduces mouse embryo viability [33]. Conversely, biopsies of bovine embryos at the pre-compacted morula stage did not adversely affect *in vitro* developmental potential, and that the morula stage was the best stage for blastomere removal [34]. The loss of viability observed when biopsies were performed at the compact morula-stage in murine embryos might be caused by cell–cell adhesion that characterizes this developmental stage. The presence of tight intercellular junctions is one reason why biopsies fail in 4-day-old human embryos [23]. We suggested performing compact-stage embryo biopsy after cell adhesions have been eliminated by artificial decompaction. The high blastocyst formation rate observed after using decompacted human morula biopsy in this study is in agreement with data obtained using pre-compacted bovine morula biopsy [34]. Whereas embryo biopsy on earlier non-compacted stage (≤8 blastomere) human [17] and bovine [34] embryos reduces viability, this might have been a consequence of the small number of cells available at the cleavage stage.

Although morula stage biopsy may result in the partial loss of cells required to form the inner cell mass (ICM) the relationship between ICM cell differentiation at the morula-stage and ICM development is not studied enough for the present. However different mammalian embryos have demonstrated a high ability for embryonic regulation at this stage. For example, microsurgical splitting of bovine, goat, porcine, and murine morulae does not adversely affect embryonic development or birth rate [35], [36], [37], [38], [39].

Compact stage embryos with severe genetic abnormalities are sorted out by natural selection as a result of embryonic genome activation [40], [14], [41]. In general, embryos without severe genetic abnormities can successfully pass the first crucial stage of early embryogenesis (i.e., the beginning of cell differentiation) [13], [14], [42]. These results confirm our data and previously published data obtained by other authors. In our study the aneuploidy rate for chromosomes X/Y in embryos biopsied on day 4 was 8,6% (56/651, Table 4), which is considerably lower than that of embryos biopsied on day 3. According to the ESHRE PGD Consortium data collection I–XI, aneuploidy of sex chromosomes is detected in 44% of embryos at the cleavage stage [5]. Furthermore, Oligonucleotide DNA Microarray analysis in blastocysts showed that 6.5% of embryos have an aneuploidy of sex chromosomes (8.6% of embryos according to our study) and 5% of blastocysts have an aneuploidy of chromosome 21 [43]. In our study, the chromosome 21 aneuploidy rate in embryos biopsied on day 4 was 5.4% (35/651, Table 4). Hence, it is important to select embryos for genetic screening after preimplantation developmental potential has been established.

We also compared several birth parameters between the two groups. However, there were no significant differences in the delivery rate, birth height, or birth weight between the groups. There were no differences in these parameters between twins and single babies. Similar results have been reported for children who were born after PGD was performed with blastomere biopsies. Therefore, compared with ICSI children, embryo biopsy at the cleavage stage does not affect the health of singleton children born after PGD or PGS [44].

This study shows for the first time that biopsy of morula-stage embryos provides sufficient cellular material suitable for genetic diagnosis regardless of which molecular genetics method is used. Mechanical cell damage is minimal, enabling nuclear fixation for FISH. There is enough time after biopsy to obtain PGD results for embryo transfer on day 5–6 in the current IVF cycle. Furthermore, most embryos reach the blastocyst stage, and the pregnancy rate is comparable to that of IVF without PGS, illustrating that development is not affected in morula-stage embryos in vitro or in vivo. To conclude, biopsy of morula-stage embryos poses no risks to the health of new-borns.
